# A comparative study of sleep and diurnal patterns in house mouse (*Mus musculus)* and Spiny mouse (*Acomys cahirinus*)

**DOI:** 10.1038/s41598-020-67859-w

**Published:** 2020-07-02

**Authors:** Chanung Wang, Lauren E. Guerriero, Dillon M. Huffman, Asmaa A. Ajwad, Trae C. Brooks, Sridhar Sunderam, Ashley W. Seifert, Bruce F. O’Hara

**Affiliations:** 1 Department of Biology, University of Kentucky, Lexington, KY 40506 USA; 2 Department of Biomedical Engineering, University of Kentucky, Lexington, KY 40506 USA; 3 Department of Neurology, Washington University School of Medicine, St. Louis, MO 63110 USA; 4 Signal Solutions LLC, 145 Graham Ave., Lexington, KY 40506 USA

**Keywords:** Non-REM sleep, Sleep

## Abstract

Most published sleep studies use three species: human, house mouse, or Norway rat. The degree to which data from these species captures variability in mammalian sleep remains unclear. To gain insight into mammalian sleep diversity, we examined sleep architecture in the spiny basal murid rodent *Acomys cahirinus*. First, we used a piezoelectric system validated for *Mus musculus* to monitor sleep in both species. We also included wild *M. musculus* to control for alterations generated by laboratory-reared conditions for *M. musculus*. Using this comparative framework, we found that *A. cahirinus*, lab *M. musculus,* and wild *M. musculus* were primarily nocturnal, but exhibited distinct behavioral patterns. Although the activity of *A. cahirinus* increased sharply at dark onset, it decreased sharply just two hours later under group and individual housing conditions. To further characterize sleep patterns and sleep-related variables, we set up EEG/EMG and video recordings and found that *A. cahirinus* sleep significantly more than *M. musculus*, exhibit nearly three times more REM, and sleep almost exclusively with their eyes open. The observed differences in *A. cahirinus* sleep architecture raise questions about the evolutionary drivers of sleep behavior*.*

## Introduction

Of the approximately 6,400 extant mammalian species^1^, sleep data has been gathered on only about 70^2^, leaving many unknowns about the variation of mammalian sleep. Of these 70 species, sleep has been detailed extensively only in three (with thousands of publications each): humans, house mice (*Mus musculus*), and Norway rat (*Rattus norvegicus*). The detailed examination of sleep architecture in other mammalian and non-model species is important to understand the common features and possible functions of sleep, as well as to identify unusual features that some species may have evolved in specific environments^3^. In order to make these comparisons between species, the common features of sleep must be noted first. Sleep has been defined with several criteria: rapid reversibility, decreased sensory responsiveness, and species-specific sleep posture^4^. This definition extends to all animals that have been determined to have sleep behaviors, not just mammals. Sleep can then be further sub-divided using physiological measures. As determined with traditional electroencephalography (EEG) and electromyography (EMG), mammals typically begin sleep with an increase in amplitude and slowing of the EEG, and a decrease in muscle tone^5^ as compared to wake, and have two very different kinds of sleep: REM (rapid-eye-movement) and non-REM (NREM). In humans, NREM is separated into various stages (N1, N2, N3), which may or may not exist in other mammals to varying degrees.

By completing comparative studies of sleep and circadian rhythms and noting unique phenomena, there are opportunities for determining the physiological basis or adaptive functions of specific sleep or circadian behaviors. To better understand sleep diversity among murid rodents, we aimed to characterize sleep and wake in *A. cahirinus*, a species in which circadian data have been collected but whose sleep behavior remains uncharacterized. *A. cahirinus* has become an important model for tissue regeneration^6^ and menstruation studies^7^, and have been shown to exhibit highly social behavior.

Spiny mice (*Acomys*), including *A. cahirinus,* live in arid areas from the Middle East to southern Asia and parts of Africa^8^. Previous circadian studies have investigated *Acomys cahirinus* and *Acomys russatus,* which when sharing a habitat have adapted to different phase preferences^9^. When isolated, both *Acomys cahirinus* and *Acomys russatus* exhibit nocturnal behaviour^10^, as expected for desert dwelling rodents, presumably to avoid intense sunlight and heat^9^. However, when the two species are sympatric, *A. cahirinus* remain nocturnal, but *A. russatus* shift their rhythm to forage during a portion of the day, becoming partially diurnal^10,11^. Field observations, trapping studies, and laboratory investigations suggest that *A. russatus* may have evolved flexible sleep patterns and a higher tolerance for heat and aridity than *A. cahirinus*^10–13^. Thus, competitive interaction may have contributed to evolutionary changes in circadian rhythms and sleep behaviors of both *Acomys* species^14^. These results may also reflect the adaptive value and a stronger role of a food-entrainable oscillator in *A. cahirinus* due to the shift in activity that corresponds to shifted foraging times^15^. A separate investigation of *A. cahirinus* rhythms used infrared motion detectors and running wheels; it also reported that *A. cahirinus* were nocturnal, with activity beginning at dark onset^16^. Under constant dark conditions, they responded with phase adjustments to light exposure as seen in other nocturnal species. These studies specifically measured waking activity, but did not investigate sleep behavior of *A. cahirinus* or any other spiny mouse species.

Here we compare sleep architecture between *A. cahirinus* and the common laboratory mouse, *Mus musculus* (specifically the outbred Swiss Webster (SW) and the inbred C57BL/6J (BL6)) as well as wild-caught *M. musculus*. The *A. cahirinus* colony at the University of Kentucky, due to their history, may retain a higher degree of variability in their behavioral and physiological characteristics than lab-maintained *Mus* strains. To assess whether variations in sleep phenotypes seen between *A. cahirinus* and Lab mice are indeed species differences, and not variations between individuals, we also included wild-caught *M. musculus*. The wild-caught *M. musculus* may contain differences between individuals similar to our sample of *A. cahirinus,* and provide a second comparison between species. To provide a detailed characterization of sleep architecture we used various methodologies: (1) a non-invasive piezoelectric system, (2) EEG/EMG, and (3) infrared (IR) cameras.

## Results

### *Acomys cahirinus* are nocturnal, but show less activity after midnight

**Figure 1:**
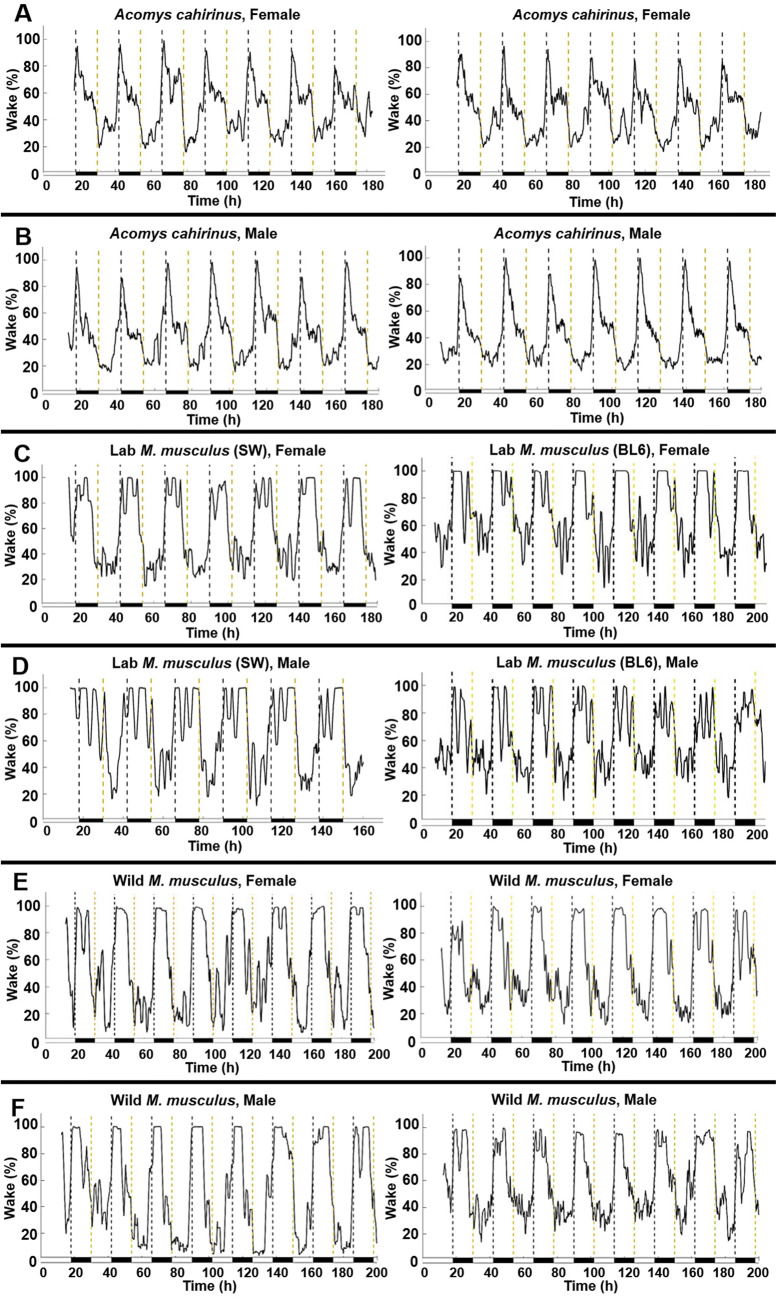
The percent wake on the Y-axis is represented as a sliding average over a 2-h window. Hours of recording are plotted on the X-axis where 0 represents midnight of day 1. Dashed vertical lines demarcate the dark phase, which is also indicated at the bottom as a heavy horizontal black line. Each two plots were shown for *A. cahirinus* and Wild *M. musculus* to show similarities of the sleep–wake profiles of two randomly selected mice in each group. One plot was shown for each Lab *M. musculus*. (**A**) *A. cahirinus* female (n = 18). (**B**) *A. cahirinus* male (n = 18). Two Lab *M. musculus*. (**C**) Lab *M. musculus* (SW) female (n = 16), Lab *M. musculus* (BL6) female (n = 9). (**D**) Lab *M. musculus* (SW) male (n = 16), Lab *M. musculus* (BL6) male (n = 9). (**E**) Wild *M. musculus* female (n = 11). (**F**) Wild *M. musculus* male (n = 11).

Using the PiezoSleep System, we recorded sleep behaviors of *A. cahirinus,* Lab *M. musculus* (SW and BL6)*,* and wild *M. musculus* for 6–8 days. This well-validated PiezoSleep system uses a non-invasive piezoelectric film placed across the cage floor that measures pressure changes due to the movement of the animal. During wake, this consisted of locomotion, rearing, postural adjustments, eating, drinking, grooming, and other behaviors, whereas during sleep the dominant signal was only due to breathing movements; this allows for sleep/wake differentiation as described by Mang et al.^30^ Using the PiezoSleep data, it was confirmed that all groups were primarily nocturnal (Fig. 1; Table 1). However, differences can be seen in the distribution of activity between species. *A. cahirinus* showed a sharp increase in activity at dark onset, which is common in nocturnal species, but then exhibited an unusual sharp decrease in activity just two hours later (Fig. 1A, B). Both inbred and outbred Lab *M. musculus* (SW and BL6) exhibited more prolonged activity across most of the night (Fig. 1C, D). Also strongly nocturnal, wild *M. musculus* displayed increased activity during most of the night, but showed a sharp decrease in activity 3–4 h before light onset (Fig. 1E, F). This drop-in activity of wild *M. musculus* was similar to the drop-in activity seen in *A. cahirinus,* but took place later during the dark phase. Given the similarities in the two lab *M. musculus* strains (SW and BL6), we discuss these together as ‘Lab *M. musculus*’. Detailed results are provided for both Lab *M. musculus* strains (Tables 1 and 2, and Supplementary Table 1), and in several figures.

**Table 1:** Piezoelectrically defined sleep amounts (% of total recording time) and sleep bout length (sec) in Lab *M. musculus*, wild *M. musculus*, and *A. cahirinus.*

Species	Sex	Interval		
		24-h	Dark	Light
**Percent (%)**				
*Lab Mus*	All	37.4 ± 2.2	19.2 ± 2.6	55.6 ± 3.4
	Male	36.8 ± 2.1	19.1 ± 2.5	54.5 ± 2.9
	Female	38.0 ± 2.2	19.2 ± 2.8	56.7 ± 3.6
*Wild Mus*	All	45.7 ± 5.5**	23.9 ± 5.8**	67.6 ± 8.2**
	Male	47.6 ± 5.2**	24.5 ± 6.6**	70.7 ± 8.7**
	Female	43.9 ± 4.8**	23.4 ± 5.1	64.4 ± 6.8**
*Acomys*	All	49.0 ± 3.4**++	34.8 ± 4.8**++	63.3 ± 2.6**++
	Male	49.6 ± 3.8**	35.3 ± 4.8** ++	63.9 ± 1.8**++
	Female	48.4 ± 3.7**+	34.2 ± 5.0** ++	62.7 ± 3.2**
Bout Length (sec)				
*Lab Mus*	All	269.0 ± 46.8	191.5 ± 36.6	312.1 ± 64.3
	Male	282.3 ± 41.3	200.3 ± 34.0	325.8 ± 65.3
	Female	255.8 ± 49.1	182.7 ± 37.7	298.4 ± 61.4
*Wild Mus*	All	442.4 ± 88.1**	257.2 ± 61.9**	608.0 ± 156.5**
	Male	469.1 ± 94.2**	264.1 ± 72.2**	656.9 ± 179.5**
	Female	415.7 ± 76.5**	250.2 ± 52.2**	559.1 ± 117.9**
*Acomys*	All	320.7 ± 35.0**++	221.5 ± 32.4**++	413.0 ± 30.4**++
	Male	332.3 ± 44.8*++	231.1 ± 36.1	411.1 ± 31.7**++
	Female	309.1 ± 30.3*++	212.1 ± 26.9	408.9 ± 29.3**++

The percentage of sleep and sleep bout length were determined for Lab *M. musculus* (n = 54), wild *M. musculus* (n = 22), and *A. cahirinus* (n = 36) using the piezoelectric system. All values are % of total recording time. *A. cahirinus* sleep significantly more during all phases. Values represent mean ± S.D

*P < 0.05, **P < 0.01 Lab *Mus* vs. *Acomys* and Lab *Mus* vs. Wild *Mus,* ^+^*P* < 0.05, ^++^*P* < 0.01, Wild *Mus* vs. *Acomys*, One-way ANOVA, Tukey's post-hoc test.

**Table 2:** Percent time spent awake in 1-h bins in Lab *M. musculus*, wild *M. musculus*, and *A. cahirinus* over 24 h.

Time intervals	Species			Male			Female		
	*Lab Mus*	*Wild Mus*	*Acomys*	*Lab Mus*	*Wild Mus*	*Acomys*	*Lab Mus*	*Wild Mus*	*Acomys*
**Light**									
6:00–7:00	64.5 ± 8.8	34.2 ± 8.9**	35.5 ± 3.5**	69.7 ± 6.8	33.8 ± 7.7**	34.4 ± 3.4**	59.2 ± 7.4	34.6 ± 10.3**	36.6 ± 3.2**
7:00–8:00	52.5 ± 7.5	31.3 ± 9.4**	28.8 ± 4.1**	54.1 ± 7.1	28.3 ± 8.0**	28.8 ± 4.0**	50.8 ± 7.6	34.3 ± 10.2**	28.9 ± 4.4**
8:00–9:00	42.5 ± 5.9	32.1 ± 9.0**	30.6 ± 3.5**	42.4 ± 6.9	28.3 ± 7.9**	30.7 ± 2.9**	42.7 ± 4.8	35.9 ± 8.8	30.6 ± 4.1**
9:00–10:00	37.8 ± 4.5	31.9 ± 8.3**	31.8 ± 3.7**	37.9 ± 4.3	26.8 ± 6.8**	31.0 ± 3.4*	37.7 ± 4.8	36.9 ± 6.5	32.7 ± 3.9
10:00–11:00	34.7 ± 4.5	28.9 ± 8.2**	32.5 ± 4.3	33.7 ± 4.1	23.4 ± 5.9**	31.7 ± 3.3+	35.7 ± 4.7	34.3 ± 6.4	33.4 ± 5.1
11:00–12:00	34.1 ± 5.1	27.0 ± 8.7**	33.5 ± 4.0++	34.2 ± 5.0	21.9 ± 6.9**	32.1 ± 2.8++	34.1 ± 5.4	32.1 ± 7.3	34.9 ± 4.7
12:00–13:00	36.2 ± 5.2	26.3 ± 9.3**	34.8 ± 3.5++	36.0 ± 4.0	21.4 ± 6.7**	33.5 ± 3.4++	36.4 ± 6.2	31.2 ± 9.2	36.1 ± 3.2
13:00–14:00	38.5 ± 6.7	26.7 ± 9.6**	35.5 ± 3.1++	38.8 ± 6.4	24.6 ± 9.2**	34.6 ± 2.6++	38.1 ± 7.2	28.8 ± 10.0	36.4 ± 3.4
14:00–15:00	39.9 ± 8.3	29.9 ± 14.2**	37.0 ± 3.7++	40.8 ± 8.3	31.2 ± 17.6**	35.2 ± 3.3	39.0 ± 8.3	28.6 ± 10.3**	38.8 ± 3.2
15:00–16:00	43.8 ± 8.8	31.7 ± 14.8**	37.0 ± 3.4**+	45.2 ± 10.3	32.3 ± 19.2**	35.7 ± 2.7**	42.5 ± 6.8	31.0 ± 9.5**	38.3 ± 3.6
16:00–17:00	47.0 ± 7.9	36.5 ± 13.3**	40.6 ± 4.6**	50.1 ± 8.0	33.0 ± 15.0**	42.3 ± 4.4**+	43.9 ± 6.4	40.0 ± 10.9	38.9 ± 4.2
17:00–18:00	61.0 ± 5.8	53.1 ± 14.0**	62.9 ± 5.8++	62.6 ± 5.3	46.8 ± 13.3**	63.3 ± 4.7++	59.5 ± 6.0	59.4 ± 12.0	62.5 ± 6.9
**Dark**									
18:00–19:00	84.7 ± 3.7	78.8 ± 8.6**	90.8 ± 2.9**++	84.8 ± 3.9	74.9 ± 8.1**	92.1 ± 2.5**++	84.5 ± 3.6	82.7 ± 7.4	89.5 ± 2.7
19:00–20:00	89.9 ± 4.4	92.7 ± 4.9	91.3 ± 3.8	90.2 ± 3.9	91.4 ± 5.4	92.9 ± 3.0	89.7 ± 5.0	93.9 ± 4.1	89.7 ± 3.9
20:00–21:00	86.6 ± 7.3	91.4 ± 6.7*	78.3 ± 8.2**++	86.1 ± 7.4	89.9 ± 7.1	83.3 ± 5.5	87.1 ± 7.4	92.8 ± 6.3	73.2 ± 7.4**++
21:00–22:00	85.3 ± 6.8	88.0 ± 8.5	69.0 ± 7.0**++	85.3 ± 6.2	85.8 ± 9.6	69.3 ± 7.1**++	85.3 ± 7.5	90.3 ± 6.9	68.7 ± 7.1**++
22:00–23:00	82.8 ± 5.9	84.9 ± 10.4	63.3 ± 5.6**++	82.7 ± 5.4	81.8 ± 12.4	63.7 ± 4.8**++	83.0 ± 6.5	88.1 ± 7.1	62.9 ± 6.4**++
23:00–0:00	82.2 ± 6.4	84.5 ± 12.9	59.5 ± 5.6**++	82.1 ± 7.5	81.6 ± 15.9	59.8 ± 5.0**++	82.3 ± 5.1	87.4 ± 8.8	59.3 ± 6.3**++
0:00–1:00	82.7 ± 6.1	83.5 ± 11.0	58.8 ± 6.1**++	82.4 ± 6.6	81.1 ± 13.2	56.5 ± 5.3**++	82.9 ± 5.7	85.9 ± 8.2	61.2 ± 6.0**++
1:00–2:00	80.3 ± 6.8	77.9 ± 11.3	58.4 ± 7.9**++	79.7 ± 7.5	77.1 ± 12.4	55.3 ± 6.1**++	80.9 ± 6.1	78.7 ± 10.6	61.5 ± 8.5**++
2:00–3:00	76.5 ± 7.2	69.1 ± 11.2**	57.5 ± 8.2**++	75.2 ± 8.4	72.3 ± 11.2	54.0 ± 7.3**++	77.8 ± 5.6	65.9 ± 10.8**	61.0 ± 7.8**
3:00–4:00	74.4 ± 6.6	60.3 ± 9.7**	56.7 ± 8.3**	73.8 ± 7.3	64.7 ± 8.4**	53.2 ± 8.1**++	75.0 ± 6.0	56.0 ± 9.2**	60.1 ± 7.1**
4:00–5:00	72.5 ± 7.6	55.6 ± 7.8**	54.2 ± 6.8**	72.5 ± 7.2	57.6 ± 7.7**	52.2 ± 7.1**	72.5 ± 8.2	53.5 ± 7.7**	56.2 ± 6.1**
5:00–6:00	72.1 ± 8.0	46.0 ± 8.6**	45.0 ± 5.6**	76.0 ± 5.8	47.8 ± 8.0**	44.0 ± 4.8**	68.2 ± 8.1	44.2 ± 9.1**	46.1 ± 6.3**

Percentage of wake averaged for each hour of the day, by population and by sex**.** Both are nocturnal species, with percentage of wake increasing sharply at dark onset. Notably, *A. cahirinus* have high wake values (over 80%) for only two hours after dark onset LD 12:12. Values represent percentage mean ± SD.

*P < 0.05, **P < 0.01, SW *Mus* vs. *Acomys* or SW *Mus* vs. Wild *Mus.* ^+^*P* < 0.05, ^++^*P* < 0.01, *Acomys* vs. Wild *Mus.*

Of all groups studied, *A. cahirinus* were found to sleep the most during the 24-h cycle, with more sleep as compared to Lab *M. musculus* (49% vs. 37.4%) and a smaller but significant increase relative to wild *M. musculus* (49% vs. 45.7%) (Table 1, with significance thresholds). *A. cahirinus* exhibited more sleep during both phases than the Lab *M. musculus* (34.8% vs. 19.2% during the dark phase and 63.3% vs. 55.6% during the light phase). Wild *M. musculus* had spent more time asleep than Lab *M. musculus* (45.7% vs. 37.4%). Of all groups, the wild *M. musculus* were found to sleep the most during the light phase. Interestingly, *A. cahirinus* spent slightly less time asleep during the light phase than wild *M. musculus* (63.3% vs. 67.5%). Dark phase activity was generally similar between the three groups of *M. musculus* (Lab (SW and BL6) and Wild). During all intervals, *A. cahirinus* showed longer sleep bouts than Lab *M. musculus* (q(105) = 5.583, *P* = 0.0004), but wild *M. musculus* exhibited the longest bouts (q(105) = 11.85, *P* < 0.0001). Also of note, there was a larger variation seen in wild *M. musculus* during all phases, as expected of a wild-caught sample (Table 1).

**Figure 2:**
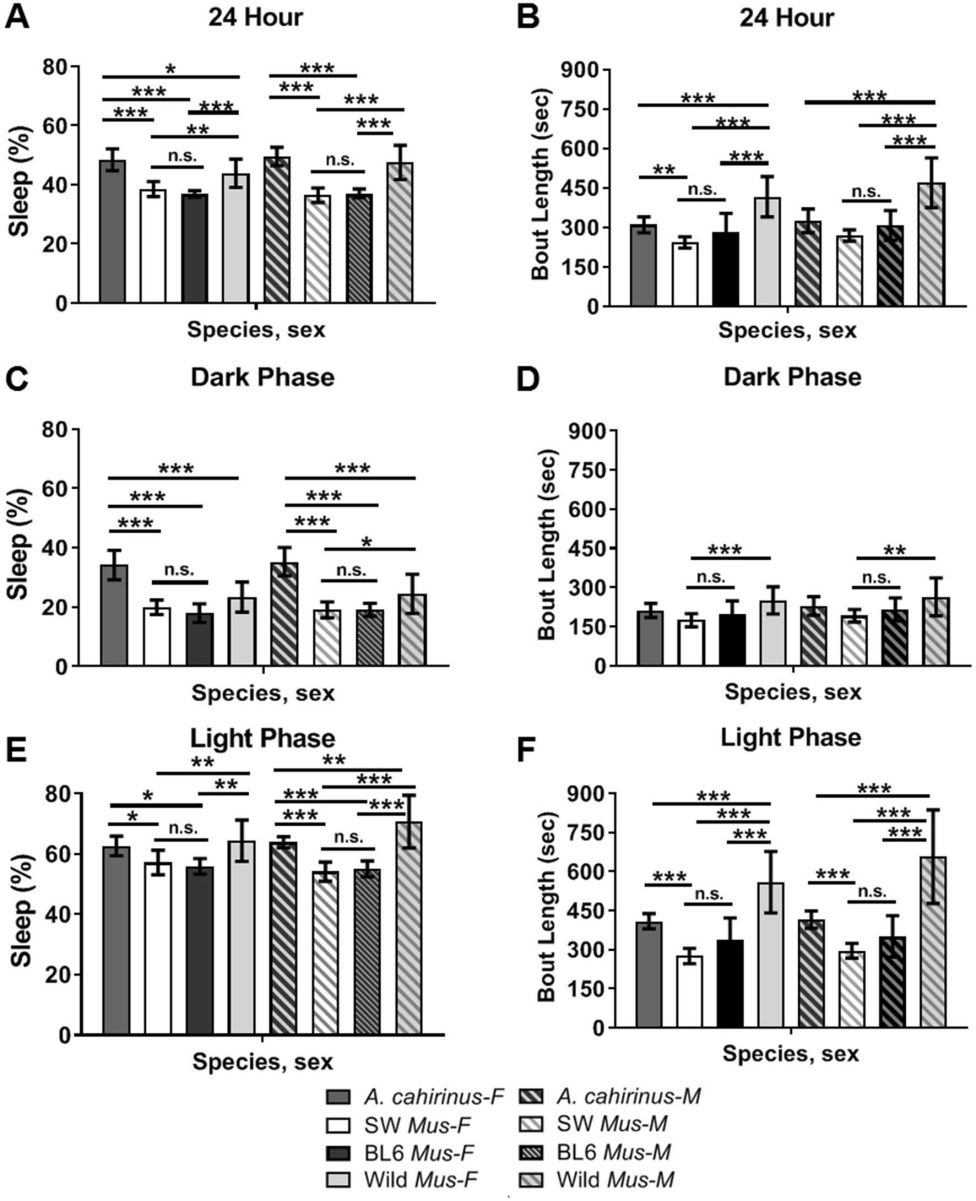
Sleep–wake patterns in *A. cahirinus*, Lab *M. musculus* (SW), Lab *M. musculus* (BL6), and wild *M. musculus.* (**A**, **C**, **E**) Average sleep times as percentages for each species and sex during (**A**) 24 h, (**C**) the dark phase, and (**E**) the light phase. (**B**, **D**, **F**) Depict average bout length in seconds during (**B**) 24 h, (**D**) the dark phase, and (F) the light phase. *A. cahirinus* (n = 36, 18 males), LAB SW *M. musculus* (n = 32, 16 males), LAB BL6 *M. musculus* (n = 18, 9 males), and wild *M. musculus* (n = 22, 11 males). Values represent mean ± SD. (*P < 0.05, **P < 0.01, ***P < 0.001. One-way ANOVA analysis:Tukey's post-hoc test).

To determine if there was variation between males and females, sex differences in activity were analyzed for each group. There were no significant differences in percent sleep time between the sexes of *A. cahirinus* (q(102) = 1.494, *P* = 0.8973), Lab *M. musculus* (q(102) = 1.76, *P* = 0.8137), and wild *M. musculus* (q(102) = 3.6, *P* = 0.1207). Once again, both *A. cahirinus* sexes were found to sleep significantly more throughout the 24-h day than Lab *M. musculus* (female, q(102) = 13.96, *P* < 0.0001; male, q(102) = 17.18, *P* < 0.0001) (Fig. 2A).

During the dark phase, sleep percentages of both female and male *A. cahirinus* were still significantly higher than Lab *M. musculus* (female, q(102) = 16.1, *P* < 0.0001; male q(102) = 17.39, *P* < 0.0001). Dark phase sleep percentages for *A. cahirinus* were also higher than wild *M. musculus* (female, q(102) = 9.362, *P* < 0.0001; male q(102) = 9.36, *P* < 0.0001) (Fig. 2C). During the dark phase, there were no significant bout differences between groups (*Acomys* vs Lab or wild *Mus*) (Fig. 2D). There were, however, significant differences in sleep bouts between Lab and wild *M. musculus* (female, q(102) = 6.357, *P* = 0.0003; male, q(102) = 6.01, *P* = 0.0007).

During the light phase, sleep time percentages for both female and male *A. cahirinus* were significantly higher than Lab *M. musculus* (female, q(102) = 6.26, *P* = 0.0003; male, q(102) = 9.807, *P* < 0.0001). Male wild *M. musculus* slept significantly more than the other groups (Lab *M. musculus*, q(102) = 14.44, *P* < 0.0001; *A. cahirinus*, q(102) = 5.730, *P* = 0.0014). Female, wild *M. musculus* also slept significantly more during the light phase than female Lab *M. musculus* (q(102) = 6.863, *P* < 0.0001), but not more than the *A. cahirinus* females (Fig. 2E). Over 24-h and the light phase, both female and male *A. cahirinus* showed longer sleep bouts than Lab *M. musculus* (female, q(102)-4.528, *P* = 0.219; male, q(102) = 4.333 *P* = 0.0324) (Fig. 2B). However, wild *M. musculus* had longer bouts than Lab *M. musculus,* and had large variations in bout lengths between individuals.

When the proportion of sleep/wake (in hour bins) was determined, the time of day significantly affected sleep–wake behavior (ANOVA: F (23, 2,520) = 802.3, *P* < 0.001; Table 2). For both sexes of *A. cahirinus,* decreased activity was seen two hours after dark onset, which is likely to be a species-specific behavior. This dip in activity was continuously lower than the activity of SW *Mus* for the rest of the dark phase [00:00–06:00]. In male *A. cahirinus,* this decrease in activity early in the dark phase was more pronounced than in females. Lab *M. musculus* showed no sex differences, with both females and males being highly active during late night periods [00–03] and across the entire dark period, with many time points of 100% wake (Fig. 1C, D).

Group differences were observed during the dark phase. At 20:00 there was a decrease in *A. cahirinus* activity, which significantly differed from both Lab and wild *M. musculus* groups (Lab *Mus*, q(2,520) = 7.516 *P* < 0.0001, wild *Mus*, q(2,520) = 9.526, *P* < 0.0001) (Table 2). Daily sleep percent analyses within each species showed that there were no sex differences. But when the timing of sleep was considered, sex differences became evident. Male *A. cahirinus* showed a larger decrease in activity after midnight than females (Table 2). Male *A. cahirinus* also slept significantly more than male Lab *M. musculus* and wild *M. musculus* (Lab, q(2,448) = 17.14, *P* < 0.0001, wild, q(2,448) = 13.15, *P* < 0.0001)*.* When comparing groups during the light phase, with both sexes combined, Lab *M. musculus* and wild *M. musculus* had significantly different percent times awake Lab *Mus*, q(2,520) = 21.49, *P* < 0.0001, wild *Mus*, q(2,520) = 17.58, *P* < 0.0001). This effect was due to differences in males, with male wild *M. musculus* spending more time awake during the light phase than male Lab *M. musculus* (q(2,448) = 8.44, *P* < 0.0001).

### Cosinor analysis confirm species differences seen in wake

**Figure 3:**
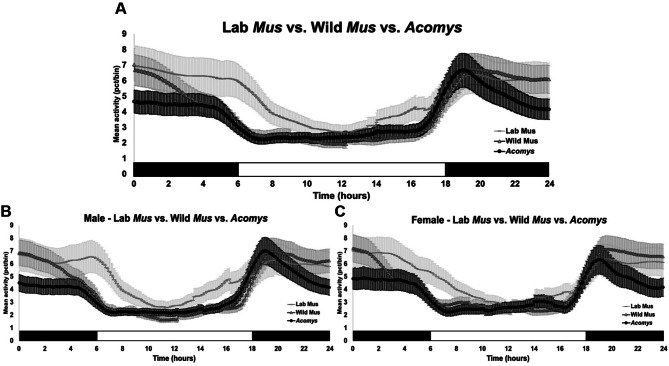
Cosinor analysis of wake percentage in *A. cahirinus,* Lab *M. musculus* (SW)*,* and wild *M. musculus.* Cosinor analysis of the activity counts gathered by the piezoelectric system. (**A**) Comparison of species, Lab *M. musculus* (SW)*,* wild *M. musculus,* and *A. cahirinus.* (**B**) Comparison of males. (**C**) Comparison of females. Values represent mean amplitude of wake percent (in 3 min bins) ± SEM.

Cosinor analysis was done to confirm the rhythms seen in the sleep–wake profiles of *A. cahirinus,* Lab *M. musculus* (SW)*,* and wild *M. musculus* (Fig. 3A–F). Results from wake percent cosinor analysis showed that the rhythm of *A. cahirinus* has a lower MESOR (midline estimating statistic of rhythm) than both SW *M. musculus* and wild *M. musculus,* especially after midnight. This suggests that *A. cahirinus* have less wake on average than the other groups (Fig. 3A). At 7:00 (light onset) until 10:00, *A. cahirinus* showed a decrease in percent wake and slept more than SW *M. musculus*. However, both *A. cahirinus* and wild *M. musculu*s showed similar amplitudes of their sleep percent rhythms around light onset and shortly after. Between species, small sex differences were seen (Fig. 3B, C). Notably, both sexes of *A. cahirinus* showed a decrease in activity after midnight, but this was more pronounced for males. This trend holds true when the data were divided between the sexes (Supp. Fig. 2) and showed hourly differences in percent wake. The average amplitude across 24 h in female *A. cahirinus* was significantly lower than both female SW and wild *M. musculus* (q(74) = 6.18, *P* < 0.001; q(74) = 9.41, *P* < 0.001). The average MESORs across 24 h in both female and male *A. cahirinus* were significantly lower than both SW and wild *M. musculus* (female, q(74) = 12.18, *P* < 0.001, q(74) = 5.87, *P* = 0.0012; male, q(74) = 13.83, *P* < 0.001, q(74) = 16.51, *P* < 0.001) (Supp. Fig. 2).

### *A. cahirinus* and *M. musculus* sleep differently in groups

**Figure 4:**
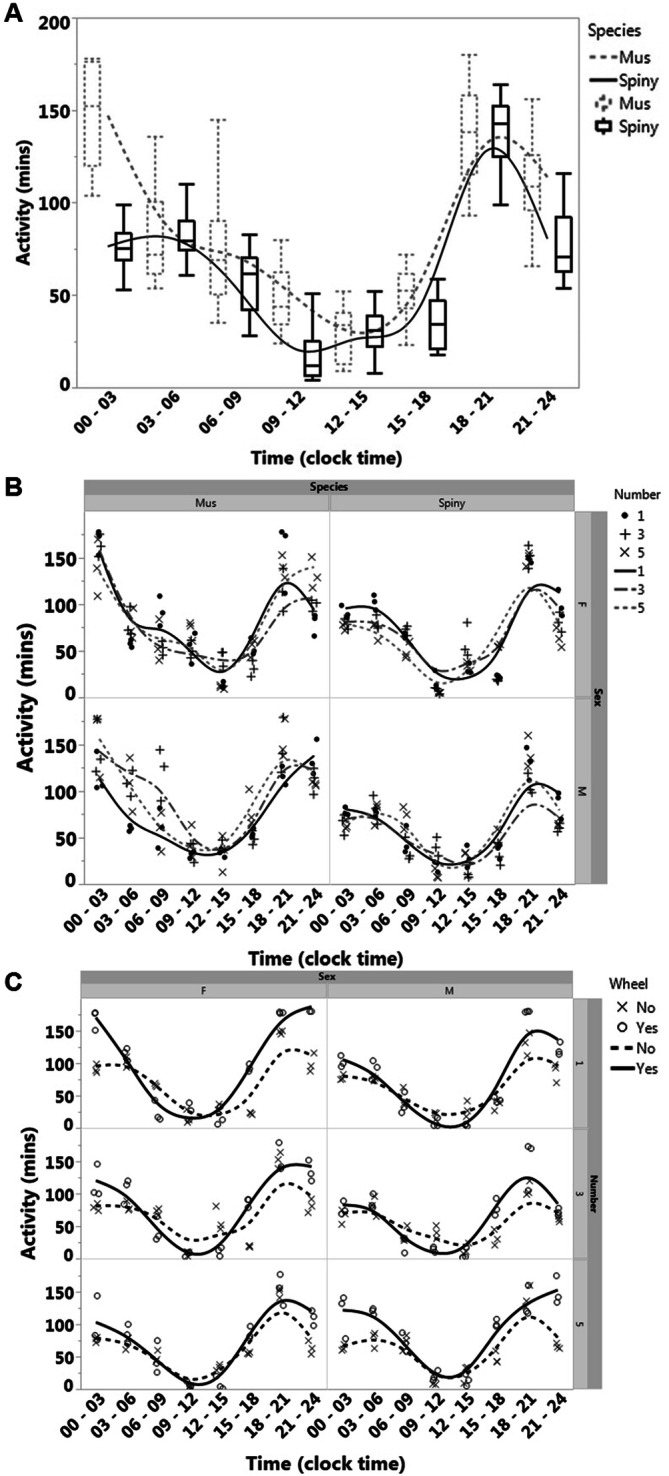
IR camera recording results. (**A**) Comparison between *A. cahirinus* and Lab *M. musculus* (SW). *A. cahirinus* are more active during early night [18–21] than late night [00–03] (Student t-test, and One-way ANOVA analysis:Tukey's post-hoc test). (**B**) The activity plotted on the Y-axis is represented as movement time during each time period, following a single animal in group conditions or alone. (C) Comparisons of *A. cahirinus* activity with or without running wheel in group conditions or alone.

*A. cahirinus* maintained in our colony exhibit highly social behaviors and under single-housed conditions often appear lethargic^8^. This has been reported previously as a common behavior of this species^8^. Therefore, in order to test for differences in sleep and in circadian patterns between group-housing and single-housing of *A. cahirinus*, we set up four IR cameras surrounding cages to get multiple angles of each condition. For individually housed conditions, IR camera data (Fig. 4A) replicated Piezo data (Fig. 1) showing that *A. cahirinus* had a sharp decrease in activity two hours after dark onset. By comparing different times during the dark phase (“early night”^18–21^ and “late night” [00–03]) we confirmed that *A. cahirinus* were more active during the early night than during late night (Fig. 4A). Conversely, the activity of SW *M. musculus* was not different during these times.

Visualizing the effect of social group interactions was done by following a single marked animal, and plotting the activities of individuals of both species and both sexes under each condition (Fig. 4B). When comparing within each species, there were sex differences in activity over all time points between *A. cahirinus* females and males, regardless of group size. SW *M. musculus* did not exhibit these sex differences. Although we only tracked the activity of the first animal, by counting activity of this marked animal, all other animals in the cage showed similar behavior (data not shown). When comparing the same sex between species, the activities of the males differed between *A. cahirinus* and SW *M. musculus*. Females, on the other hand, had highly variable activity (some days females were highly active and other days would be sedentary) and there appeared to be no significant differences between species (unpublished observations).

To understand the effect of group size on activity levels, we tested different same-sex groupings (1, 3, and 5 animals). Group size affected female *A. cahirinus* activity at [00–03] and [06–09] (Fig. 4B). The more female spiny mice present, the lower the overall activity. In male *A. cahirinus,* this effect was similar but less pronounced with larger numbers having less activity. Both female and male SW *M. musculus* had no changes in activity due to group size. Using these results, we concluded that the activity of *A. cahirinus* during late night differs from both the activity of SW *M. musculus* at the same time, and the activity of *A. cahirinus* during other time periods.

While using the IR cameras, we also investigated the impact of running wheels on group activity of *A. cahirinus.* Using groups of 1, 3, and 5 same-sex mice, we recorded using the same methodology with the addition of a running wheel. According to our observations, the added running wheel did not change the amount of activity in the different group conditions but changed the timing of activity (Fig. 4C). Male *A. cahirinus* were more active with the wheel during late night in a group of 5, than in smaller groups or when alone. The females, on the other hand, were more active with the wheel during late night when alone, than when in groups of 3 or 5 (Fig. 4C).

### EEG profiling of *A. cahirinus* show they exhibit significantly more REM compared to *M. musculus*

Analysis of piezo data showed relatively high amounts of total sleep in *A. cahirinus*. To investigate this in greater detail, a subset of male *A. cahirinus* were assessed by traditional EEG/EMG methods. EEG/EMG recordings were done in conjunction with Piezo recordings, which provided valuable information for state determination. The EEG signals of *A. cahirinus* were typical of sleep and wakefulness in other rodents^20^, with wake marked with higher frequency and variable amplitude signals and sleep clearly divided into NREM sleep marked with slower frequency waves (0.5–4.0 Hz) at high amplitudes (typical of mammalian Slow-wave Sleep (SWS)), which was interrupted by periods of REM characterized by higher theta activity (4-7 Hz) and very low or nearly flat EMG (except for occasional twitches).

**Figure 5:**
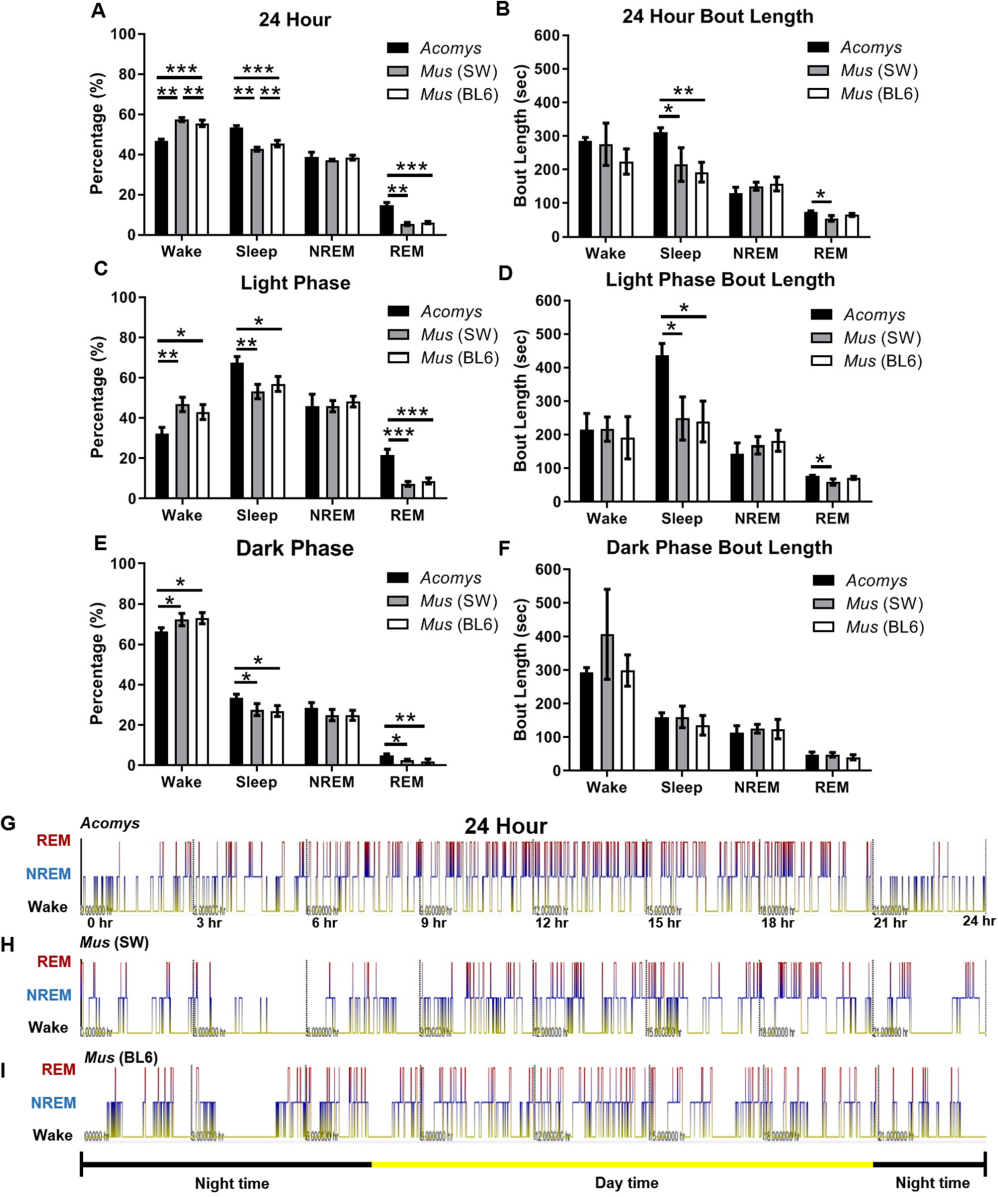
EEG profiles of male *A. cahirinus* vs. Lab *M. musculus.* (**A**) Daily percentages of vigilance states. (**B**) Average bout lengths over 24 h. (**C**) Percentage of vigilance states during the light phase [07–21]. (**D**) Average bout lengths during the light phase. (**E**) Percentage of vigilance states for the dark phase [21–07]. (**F**) Average bout lengths during the dark phase. (**G**) Hypnogram from a single *A. cahirinus* male, showing the transitions between wake (yellow, bottom), NREM (blue, middle), and REM (red, top). The horizontal bar at the bottom indicates the light phase (yellow) and dark phase (black). (H) Hypnogram from a single Lab *M. musculus* male (SW). (I) Hypnogram from a single Lab *M. musculus* male (BL6). Values represent mean ± SD. (*P < 0.05, **P < 0.01, ***P < 0.001. One-way ANOVA analysis:Tukey's post-hoc test).

According to this study, *A. cahirinus* spent 46.6% of their time awake, 38.7% in NREM sleep, and 14.7% in REM sleep (Fig. 5A). Wake for *A. cahirinus* was significantly less than SW *M. musculus* (q(7) = 21.96, *P* < 0.0001) and BL6 *M. musculus* (q(7) = 17.39, *P* < 0.0001) showing consistency with the PiezoSleep study. *A. cahirinus* had longer sleep bouts (309 s) than SW *M. musculus* (215 s) (q(7) = 4.796, *P* = 0.0275) and BL6 *M. musculus* (192 s) (q(7) = 6.363, *P* = 0.0069) (Fig. 5B). NREM sleep percentage was similar for *A. cahirinus* (38.7%)*,* SW *M. musculus* (37.2%), and BL6 *M. musculus* (39.4%)*,* while the amount of REM was significantly higher in *A. cahirinus* (14.7%) than SW *M. musculus* (5.4%) (q(7) = 17.01, *P* < 0.0001) and BL6 *M. musculus* (6%) (q(7) = 17.01, *P* < 0.0001). Also, *A. cahirinus* had longer REM bouts (73.6 s) than SW *M. musculus* (54.7 s) (q(7) = 5.62, *P* = 0.013 and BL6 *M. musculus* (65 s) (q(7) = 2.728, *N.S.*).

As expected for nocturnal animals, sleep percentage for *A. cahirinus* (67.6%) increased during the light phase (Fig. 5C) to an extent that was significantly higher than that seen in SW *M. musculus* (53.2%) (q(7) = 7.158, *P* = 0.0036) and BL6 *M. musculus* (57%) (q(7) = 5.633, *P* = 0.0128). This difference between species was due to *A. cahirinus* having longer sleep bouts (437 s) than SW *M. musculus* (248 s) (q(7) = 5.858, *P* = 0.0105) and BL6 *M. musculus* (239 s) (q(7) = 6.563, *P* = 0.0058) (Fig. 5D) and *A. cahirinus* having more REM (21.6%) than SW *M. musculus* (7.3%) (q(7) = 12.74, *P* < 0.0001) and BL6 *M. musculus* (8.8%) (q(7) = 12.19, *P* < 0.0001); *A. cahirinus* also had longer REM bouts (q(7) = 5.442, *P* = 0.0152).

The active phase for both species, the dark phase, showed an increase in wake percent (Fig. 5E). *A. cahirinus* (66.3%) spent significantly less time awake time than SW *M. musculus* (72.5%) (q(7) = 4.173, *P* = 0.0496) and BL6 *M. musculus* (73%) (q(7) = 4.821, *P* = 0.0268). Also, there was significantly more REM in *A. cahirinus* (4.9%) than in SW *M. musculus* (2.5%) (q(7) = 4.809, *P* = 0.0271) and BL6 *M. musculus* (2.1%) (q(7) = 5.998, *P* = 0.0093). There were no differences between bout lengths of any state during the dark phase (Fig. 5F).

The hypnograms show one representative individual of the frequent vigilance state changes in *A. cahirinus* (Fig. 5G) and display more wake than seen in SW *M. musculus* and BL6 *M. musculus* (Fig. 5H). Hypnograms of other individuals matched this pattern. During the light phase specifically, SW *M. musculus* and BL6 *M. musculus* had shorter sleep bouts and less REM than *A. cahirinus.* Also of note were frequent REM bouts during the light phase in *A. cahirinus* which resulted in the species’ high amount of REM sleep across the full 24 h day.

**Figure 6:**
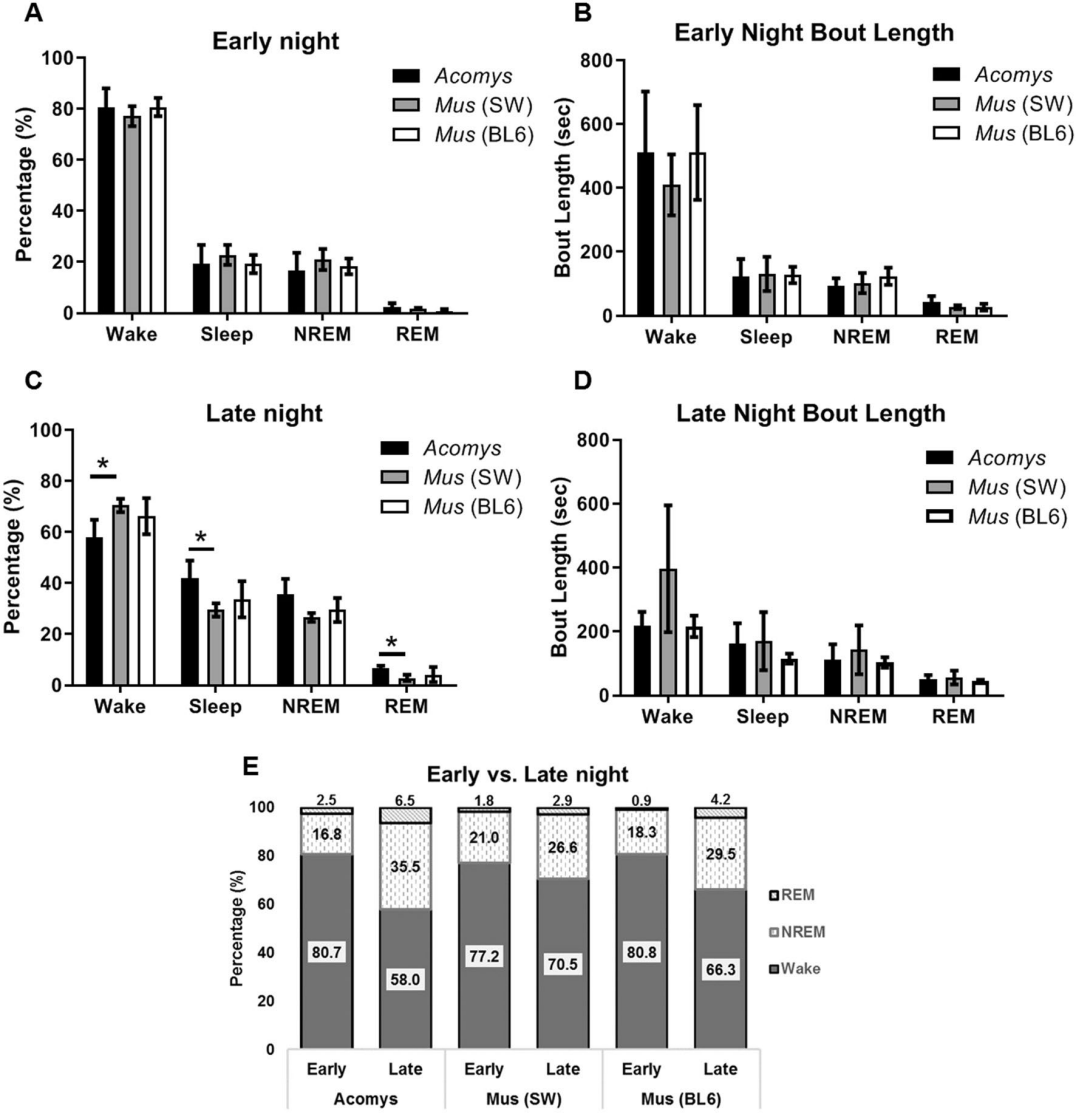
EEG profiles for both *A. cahirinus* and Lab *M. musculus* during selected times of the dark phase. (**A**) Percentages of vigilance states during early night [21–23:30]*.* (**B**) Average bout lengths during early night. (**C**) Percentages of vigilance states during late night [02–04:30]. (**D**) Average bout lengths during late night. (E) Comparisons of early night and late night for both species (*P < 0.05, One-way ANOVA analysis:Tukey's post-hoc test).

To follow up on the Piezo data that showed reduced activity during the late night, we analyzed EEG/EMG profiles of the dark period in more detail (Fig. 6). Early night was designated as the first quarter of the dark phase at clock time [21–23:30], and late night was designated as the third quarter of the dark phase [02–04:30]. During early night, *A. cahirinus* were awake 80.7% of the time, while NREM percentage was 16.8%, and REM percentage was 2.5%. There were no significant differences between species. In contrast, the late night period of *A. cahirinus* showed a marked decrease in wake (58%) and an increase in NREM (35.5%). There also was a significant increase in REM percentage (6.6% during late night compared to 2.5% during the early night). There were no statistical differences in bout lengths between early night and late night of *A. cahirinus.* During the early night, there were no significant differences in state percentage and bout lengths between *A. cahirinus,* SW *M. musculus,* and BL6 *M. musculus*. Differences between species were seen during the late night; SW *M. musculus* (70.7%) and BL6 *M. musculus* (66.3%) have more wake than *A. cahirinus* (58%) (SW, q(7) = 4.174, *P* = 0.0496; BL6, q(7) = 2.916, *P* = 0.1678). The increase of sleep in *A. cahirinus* was also due to more REM in *A. cahirinus* (6.5%) than SW *M. musculus* (2.9%) (q(7) = 4.179, *P* = 0.0492).

### Spectral analysis of EEG reveals interesting differences between species

**Figure 7:**
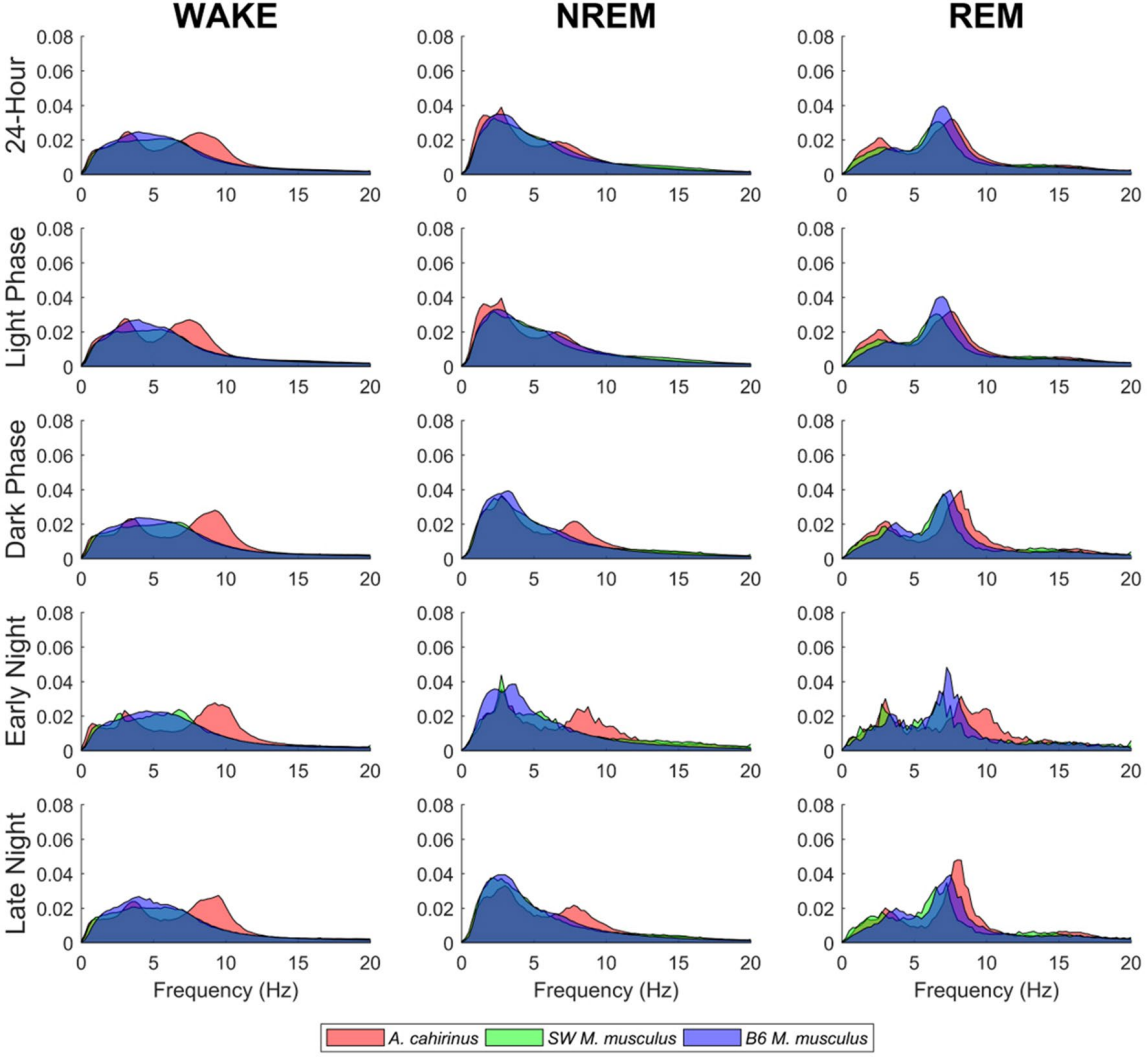
Comparison of EEG power spectra between *A. cahirinus* and Lab *M. musculus* strains*.* EEG power spectra is shown by signal power by frequency in each vigilance state (columns) and time period of the 24-h cycle (rows). Like the M. musculus strains, *A. cahirinus* shows characteristic delta and theta rhythms during NREM and REM sleep. In general, the theta rhythm seen in *A. cahirinus* is of a higher mean frequency and more prominent than in the Lab *M. musculus* strains. A strong theta component can also be seen in both Wake and NREM states, which is diminished or absent in *M. musculus*.

After observing notable differences in vigilance state cycling between *A. cahirinus* and Lab *M. musculus*, the spectral composition within each state was compared across animals, revealing time-, species- and strain-dependent differences. Both qualitative and quantitative differences can be seen in the EEG of *A. cahirinus* compared to the Lab *M. musculus* strains (Fig. 7). Like other species, *A. cahirinus* exhibited a characteristic delta rhythm during NREM sleep/SWS and higher theta rhythm during REM sleep. Interestingly, *A. cahirinus* also exhibited a higher peak in the theta band during waking and NREM states relative to both strains of Lab *M. musculus.* This theta component persists even when excluding epochs that were within 20 s of state transitions, suggesting that this is not a result of transition-related scorer error. However, we cannot be sure whether these results represent true neurophysiological differences or are a consequence of the more posterior electrode placement on *A. cahirinus*. *A. cahirinus* are larger in size than the *M. musculus* strains, so the headmount had to be moved back slightly for the EMG leads to reach the nuchal muscle.

Lastly, throughout these sleep studies using PiezoSleep based algorithms, IR video, and traditional EEG/EMG measures, we noticed that *A. cahirinus* sleep almost exclusively with their eyes open during both NREM and REM sleep. *A. cahirinus* have normal appearing eyelids, blinking responses, and are capable of eye closure during sleep, but keep their eyes open approximately 90% of all sleep periods. This was consistent under the slightly different lighting conditions used in these different studies. The different lighting conditions also did not appear to significantly influence sleep behavior as PiezoSleep, IR, and EEG/EMG studies showed quite similar sleep and wake amounts under all conditions.

## Discussion

Comparative studies describing the sleep patterns of different species may provide valuable insights for understanding the adaptive value of sleep and circadian variations. As new species are studied, more variety in sleep is discovered that may change our understanding of sleep. One such example is the dairy cow (*Bos taurus*) which undergo wake, NREM sleep, REM sleep, and an unusual, perhaps intermediate, state called drowsiness which occupies one third of their day^17,18^. By studying *A. cahirinus,* we also found species specific variations in sleep. In this study, we found basic EEG parameters to be “typical” of commonly studied laboratory rodents, but still found differences in the patterns and amounts of each arousal state, with especially high REM percent. In addition to significantly more REM sleep, we found the very unusual feature of sleep occurring with open eyes essentially 100% of the time.

Our study is the first to characterize sleep amounts and sleep architecture of *A. cahirinus*. While previous research has demonstrated that *A. cahirinus* are nocturnal with high activity consistently during the dark phase^9,15,19^, there was a lack of specific sleep data. Our data obtained using the PiezoSleep recordings demonstrated that *A. cahirinus* sleep more than SW *M. musculus* in both the dark and light phase. In the dark phase, the activity of *A. cahirinus* increased at dark onset, but surprisingly decreased after just two hours, resulting in more sleep during the dark phase. This differs from the reported activity profiles of *A. cahirinus* by Weber and Hohn^16^. They state that during individual monitoring most individuals were awake throughout the dark phase, with only a small number of spiny mice showing concentrated activity either early or late in their active phase. In contrast, our data showed that this decrease of activity two hours after dark onset is widespread in *A. cahirinus* and was consistent across our three different methods of sleep analysis. One possible explanation for this shorter period of consolidated wake is the natural high foraging ability in this species relative to other rodents. According to Gutman and Dayan^11^, the foraging efficiency of *A. cahirinus* is much greater than the closely related *A. russatus.* After foraging quickly, which is especially easy with ad libitum food in this laboratory setting, we observed *A. cahirinus* return to being more inactive/sleeping. More detailed analysis of foraging behavior in a naturalistic setting would allow for testing this hypothesis.

When considering species specific sleep patterns, our initial comparisons between SW *M. musculus,* a lab-maintained line that has been in captivity for many generations, and *A. cahirinus,* a newly established line with only a few generations in captivity, raised some concerns. For example, the lines’ history, domestication, and potential limit of genetic variations might underlie some of the differences in sleep measures rather than actual species differences. By investigating both SW *M. musculus* and wild-caught *M. musculus,* we aimed to partially address this concern. Our sample of wild *M. musculus,* while somewhat different than the lab strain, still displayed some of the species differences first noted. These wild caught mice also showed large variability in sleep phenotypes between individuals perhaps reflecting a higher genetic variation typical in most non-domesticated “wild” populations. We also had concerns that SW is an albino strain, which might differ in sleep behaviors, and thus included the most widely studied inbred strain, C57BL/6J (BL6), in many of the studies. This highly pigmented, black, inbred, laboratory strain was generally quite similar to the outbred SW strain (which was originally chosen to roughly match the genetic diversity of our *A. cahirinus* spiny mice). While albino strains could potentially be more sensitive to light or other variables, most previous studies of different mouse strains have not noted any differences correlated with albinism (e.g. see Ref.^20^, Franken et al., 1998).

The PiezoSleep system, being non-invasive, allowed for accurate recordings of all three groups, including the highly active and aggressive wild caught mice that would be more difficult to study by traditional sleep methodologies (EEG/EMG). Piezo data showed that our wild *M. musculus* slept more than SW *M. musculus*, but *A. cahirinus* showed higher amounts of sleep than both SW and wild *M. musculus*, supporting that this, at least in part, is a species-specific variation of sleep. The discrepancy seen between wild and SW *M. musculus* populations was not unexpected since many different common inbred strains of *Mus*^20^ have shown modest variations in sleep patterns. These data generally support that *A. cahirinus* have differing sleep physiology from the two samples of *Mus* studied, and due to the logistics of maintaining and studying wild caught mice, led us to focus the remainder of our studies on *A. cahirinus* and our SW *M. musculus.*

To provide a comprehensive measure of sleep architecture, sleep data was gathered using a variety of methods. Due to the nature of the PiezoSleep system, single animals had to be recorded in individual cages. While both species used in this study are social animals, observations of behavior show that *A. cahirinus* appear to be more social than *M. musculus.* During periods of low activity or during sleep in their group home cages, *A. cahirinus* sit huddled together in large groups more than SW *M. musculus*. Especially late at night [00–03], spiny mice sit together with limited movements. Because spiny mice have a propensity to maintain collective groups, this provided an opportunity to study interactions of social behavior and activity patterns^21^.

Different social conditions were investigated first using IR cameras. To study these social dynamics on the activity of *A. cahirinus,* animals were separated into different size groupings (1, 3, or 5) of the same sex. Our data showed that there was an effect of activity that depends on group size and sex. Female spiny mice in a group of 5 became significantly less active at [00–03] and [06–09] than when they are alone or in a group of 3. Males, similarly, were also less active in a larger group. This may be due to group thermoregulation, with smaller groups of spiny mice needing to stay active to maintain body temperature. Thermoregulation is often a very social activity, that is shared across many species^22^. This group activity may help reduce the energy and water costs of maintaining thermoregulation, which is important for small mammals that lose heat rapidly and need to compensate by eating large amounts of food^23^. *Acomys* show evidence of other mechanisms to prevent water loss, which is a major concern in their arid environment. They can rapidly regulate their evaporative water loss to about 14% (which is much lower than the 30–40% typical for mammals)^24^. Because the costs of thermoregulation are reduced when sharing body heat within their social group, this is also considered a possible mechanism to describe *A. cahirinus’* extremely social nature^22,23^.

In contrast, SW *M. musculus* showed no effect on activity due to number of individuals per cage. Due to this species difference, *A. cahirinus* may provide a good model to study the interactions between social behaviors, group thermodynamics, activity patterns, and sleep amount. Sleep has not been well investigated in group versus individual housing in any rodent; our limited study in these two species suggests there are not dramatic differences in total sleep times, either REM or NREM, that are dependent on social interaction. The changes we saw tended to be subtle and we are pursuing them with additional studies. Increasing recording times beyond three days may be important to observe group dynamics and could possibly show a more defined effect of group size on activity levels.

In well studied species, EEG is considered the gold-standard for determining sleep states. Our study is the first to use EEG to determine sleep states in *A. cahirinus.* While electrophysiological changes during sleep for *A. cahirinus* were generally similar to other rodents and fit the standard definitions for each arousal state^25^, spectral differences within states were noted relative to *M. musculus*. The presence of a theta peak in all states could be due to slightly different electrode placement for *A. cahirinus*, since electrodes are centered over the bregma, and *A.cahirinus* have a larger skull. This slightly more posterior electrode placement could have resulted in the EEG leads being closer to the dorsal hippocampus, and therefore able to record a stronger theta rhythm. Overall, EEG data indicated that *A. cahirinus* sleep more (53% of the total day) than SW *M. musculus* (42%). This matches other sleep studies in *M. musculus* which report sleep duration to be 36–48% of the day, which varies by strain^20^.

Another striking difference we found between species was the duration of REM sleep. REM in *M. musculus* is a small percentage of total time, only 5–6% of the day, and this duration can also vary between strain^20^. We found that *A. cahirinus* spend much more time (14% of the day) in REM sleep than SW *M. musculus,* or any other strain thus far measured^20^*.* The implications of this finding are unclear. One possible explanation is that *A. cahirinus* were able to get more REM sleep due to their social interactions and group thermoregulation. The Energy Allocation Model of Sleep states that during REM sleep thermoregulation is paused and energy is reallocated to other processes ^26^. For a very social animal, *A. cahirinus,* that share body heat with many individuals, REM sleep may take place more often because individuals need to use less energy or be less diligent in maintaining optimal thermoregulation. As Gravett and colleagues recently discovered, sociality can affect REM sleep episode duration in rock hyrax, *Procavia capensis* ^27^. They suggest increased REM duration under social conditions might better support thermoregulation strategies. The adaptive value of getting more REM is not clear, although very high REM sleep amounts are typical of mammals in prenatal and early postnatal development, and may promote higher levels of synaptic plasticity, as has been shown dramatically in cats with visual cortex remodeling following the forced closure of one eye^28^.

Investigations of sleep in new mammalian species are likely to yield new insights into fundamental questions about sleep. Some of the sleep variations in *A. cahirinus,* especially the high amount of REM, may be connected to other interesting aspects of *A. cahirinus* physiology. Fully grown *A. cahirinus* do not close their eyes during sleep, a behavior which to our knowledge, has yet to be documented for any mammalian species. Using IR camera recordings for 3 days in a row, we confirmed that *A. cahirinus* always keep their eyes open in both LD (12 h light, 12 h dark) or DD (continuous dark). EEG/EMG confirmed that *A. cahirinus* can get both REM and NREM sleep with their eyes open. This behavior is seen in healthy individuals of this species and has potential implications for understanding the effects of light and visual processing during sleep. Sleep with eyes fully or partially open occurs in many mammalian species, including sleepwalking in humans, but to our knowledge occurs only during a small portion of sleep or under unusual conditions, and has not been quantified or well-documented in any mammal.

Our characterization of sleep in *A. cahirinus* found some species-specific sleep behaviors of this spiny mouse. *A. cahirinus*, a nocturnal rodent, were found to sleep significantly more than SW *M. musculus* and wild *M. musculus*. The distribution of sleep varied between species, with Piezo data showing that *A. cahirinus* having a dramatic decrease of activity 2 h after dark onset, and sleep more later in the dark period. Social dynamics were found to impact sleep in *A. cahirinus* with groups of 5 being less active than single individuals. EEG/EMG analysis show that *A. cahirinus* get significantly more REM sleep than SW *M. musculus.* Finally, *A. cahirinus* were found to sleep with their eyes fully open. To follow up on this finding, future experiments are aimed at determining if *A. cahirinus* can process visual signals while sleeping with their eyes open and how light may affect their sleep as compared to *M. musculus,* who sleep predominately with eyes closed, or partially closed.

## Materials and methods

### Animals

*Acomys cahirinus* and *M. musculus* (Swiss Webster Envigro_Harlan Hsd:ND4 (SW) and C57BL/6J (BL6)) were housed at the University of Kentucky, Lexington, KY. *A. cahirinus* were housed at a density of 10–15 individuals per cage in metal wire cages (24 inch × 18 inch × 16 inch, height/width/depth; Quality Cage Company, Portland, OR). When maintained on high nutrient and sucrose diets, *A. cahirinus* are likely to overeat and can become spontaneously diabetic^8^. To ensure the animals were healthy and did not develop this disorder, *A. cahirinus* were fed on a diet of 3:1 mixture by volume of 14% protein mouse chow (Teklad Global 2014, Harlan Laboratories, Indianapolis, IN) and black-oil sunflower seeds (Pennington Seed Inc., Madison, GA) ad libitum^8^. SW *M. musculus* and BL6 *M. musculus* were housed at a density of 2–4 individuals per cage, in standard static microisolator cages, and were fed 18% protein mouse chow (Tekland Global 2918, Harlan Laboratories, Indianapolis, IN) only. The three populations of *M. musculus* were differentiated in this manuscript by the Swiss Webster laboratory mice referred as “Lab *M. musculus* (SW)”, the C57BL/6J laboratory mice referred as “Lab *M. musculus* (BL6)”, and the wild-caught mice referred as “wild *M. musculus*”. Lab *M. musculus* were maintained and bred in a cycle of 12 h light and 12 h dark (12:12 LD). *A. cahirinus* were bred and maintained at 25 °C on natural light (average of 200 lx) through windows in a separate humidity-controlled laboratory facility, which during this study was approximately LD 14:10, but with gradual onsets and offsets of light. This semi-natural lighting condition has been used in many previous studies of *A. cahirinus*, including comparisons to lab *M. musculus*^8,21^.

The wild mice were live trapped at the C. Oran Little Research Center, operated by the College of Agriculture at the University of Kentucky, using Sherman traps baited with oats and peanut butter. All wild mice were maintained in group housing in metal wire cages at a density of 5–12 individuals on 12:12 LD at the wild animal facility (to prevent disease transfer to the UK colony) for 3 weeks before beginning sleep recordings. Wild mice were given water and 18% protein mouse chow, ad libitum*.*

All experimental animals were acclimated to the experimental room (12:12 LD or 14:10 LD) for at least 14 days before all recordings were performed. All procedures used in the study were approved by the Institutional Animal Care and Use Committee (IACUC) of the University of Kentucky. All methods were performed in accordance with the relevant guidelines and regulations.

### Sleep and wake rhythm measurements by the piezoelectric system

Sleep and wake states were determined using a non-invasive, piezoelectric system (Signal Solutions, LLC, Lexington, KY, USA). This piezoelectric system was described in detail elsewhere^29,30^, but was comprised of plexiglass cages with a piezoelectric film lining the bottom that detects pressure variations due to the movement of the animal (Supp. Fig. 1). When the animals were sleeping, the primary pressure variations were from breathing and provide an accurate respiratory trace. Sleep states were characterized by quasi-periodic signals with low variations in amplitude. Wakefulness and rest states were characterized by irregular transient and high amplitude pressure variations corresponding to body movements and weight shifting. Even during “quiet rest”, subtle head or other movements were sufficient to distinguish rest from sleep with an accuracy comparable to EEG/EMG^30^. All animals, spiny mice (*A. cahirinus*), house mice (SW and BL6 *M. musculus),* and wild mice (*M. musculus*) were exposed to 12:12 LD for piezoelectric sleep and activity recordings. Food and water were provided ad libitum*.*

### IR recording in single and group housing cages

Four IR cameras (ZP-KE1H04-S, Zmodo Technology, China) were set up surrounding the cage to record movement. Behavior of *A. cahirinus* and SW *M. musculus* were assessed with IR camera recordings in both single and group housing conditions. All group housing experiments were conducted under semi-natural light conditions (due to windows present in this particular animal room) that were similar to 12:12 LD, (with a light period spanning from 10.5 to 12.8 h). As mentioned in results, the different lighting did not appear to alter sleep behavior or sleep amounts compared to the studies using typical artificial light with a 12:12 LD cycle. We performed IR camera recording from September (07:20 sunrise, 19:44 sunset) to early November (08:03 sunrise, 18:38 sunset). Food and water were provided ad libitum.

In all IR camera recordings, a single animal was observed alone for 4 days (1-day adjustment and 3 days for sleep quantification). Activity was manually determined for each minute bin based on whether the mouse did or did not move. Next, two additional mice of the same sex were added to form study groups of three for the next 4 days. The activity of the first mouse, distinguished with black marker on the tail, was continuously traced in all conditions. After this recording, two more mice were added, to give a total of five animals in the cage. The entire procedure required a total of 12 days and was repeated for each sex of *A. cahirinus* and SW *M. musculus*.

We also researched the effect of the running wheel on *A. cahirinus*. During a subset of the recordings, as described above, a running wheel (23 cm diameter, Pennine Metal Play Wheel, United Kingdom) was set up in the group cage without any restriction. The recorded IR videos were observed and scored manually. Three independent scorers assessed activity and behaviors in each video blinded to the others’ scores. Activity counts were grouped into three-hour bins for the entire 24 h recordings. The primary variables assessed were observation time, species (*A. cahirinus* and SW *M. musculus)*, sex (male and female), and number of mice (1, 3, and 5) in the group cage.

### EEG/EMG recording: surgery, sleep scoring, and spectral analysis

Following previously described methods^31^, a 24-h record of EEG, EMG, and Piezo signals, along with continuous video, were recorded from male *A. cahirinus,* SW *M. musculus,* and BL6 *M. Musculus.* EEG and EMG signals were preamplified (100 ×) at the head-mount and transmitted via the commutator to a 50× biosignal amplifier (8200 series, Pinnacle Technology, Inc., Lawrence, Kansas, USA). Recordings were then segmented into 4-s epochs and independently scored by experienced human raters as wake, REM (rapid eye movement), or NREM sleep. Wake was characterized by low amplitude EEG, but relatively high amplitude and variable EMG. REM was characterized by a theta EEG rhythm (6–9 Hz) and suppressed EMG (except for occasional muscle twitches). NREM, which is essentially synonymous with slow wave sleep in rodents, was characterized by low frequency, large amplitude delta EEG oscillations (0.5–4 Hz) and low tonic EMG. Video recordings were used in conjunction with EEG and EMG to validate each state when necessary. Bout length averages were compiled by the Sirenia program provided by Pinnacle Technology, which uses a conservative algorithm for bout lengths requiring sustained changes in arousal state to switch state. This produces slightly longer bout length averages than other methods, but was very consistent, and provided a good comparison across species. During EEG recordings, all animals were exposed to a 14:10 light–dark cycle for sleep and activity recordings because it closely matched the natural lighting conditions in which they were previously housed at that time of year (mid-summer). Food and water were provided ad libitum*.*

Because of the interesting behavior observed in *A. cahirinus* during the dark period, Piezo, IR, and EEG data during this time were segmented into quarters, of which the data within the first and third quarters (dubbed early night and late night) were compared. For piezoelectric recordings, early night takes place during clock time [18:00–21:00], while EEG recordings had early night during [21:00–23:30]. Late night took place during [00:00–03:00] for the piezoelectric recordings, and [02:00–04:30] for the EEG recordings. Despite different lighting conditions due to animal housing logistics, sleep and wake behaviors were quite consistent across all methods of sleep assessment (see “Results”).

EEG data (sampled at 400 Hz) was segmented into 4-s epochs (consistent with the segmentation used in manual scoring), within which the power spectral density (PSD) was estimated at 0.25 Hz resolution using the ‘periodogram’ function in MATLAB (Mathworks, Inc). PSD estimates were normalized by the total power within that epoch to allow consistent comparisons across animals/species. PSDs were then averaged separately for REM, NREM and Wake epochs in each species. Epochs for which the two manual raters disagreed on the vigilance state label, as well as those that were within 3 epochs (12 s) of a state transition were excluded from the PSD analysis.

### Statistical analysis

All statistical calculations and analyses were performed using GraphPad Prism 6 (GraphPad Software Inc, La Jolla, CA). All data were expressed as the mean ± SD (N as indicated in the figure legends). One-way analysis of variance (ANOVA) with Tukey’s multiple comparison post hoc test was performed to determine significant differences between the daily sleep profiles of the two species, and then Student’s *t* for independent measures was applied. For the comparison between species and sexes, wake percentage, averaged for each hour of the day, was analyzed using two-way ANOVA with Bonferroni's post hoc test. All results with *p* < 0.05 were considered statistically significant.

## Supplementary information

Supplementary file1 (PDF 427 kb)
