# Supplementary material for: A comparative study of sleep and diurnal patterns in house mouse (Mus musculus) and Spiny mouse (Acomys cahirinus)
Source: Sci Rep. 2020 Jul 2;10:10944. doi: 10.1038/s41598-020-67859-w (PMC7331618; doi:10.1038/s41598-020-67859-w)
Supplement: Supplementary file 1 — Supplementary file1 (PDF 427 kb) [file 41598_2020_67859_MOESM1_ESM.pdf]

Supplementary Material for

A comparative study of sleep and diurnal patterns in house mouse (*Mus musculus)* and Spiny mouse (*Acomys cahirinus*)

Chanung Wang[1,3]
Lauren E. Guerriero[1]
Dillon M. Huffman[2]
Asmaa A. Ajwad[2]
Trae C. Brooks[1]
Sridhar Sunderam[2]
Ashley W. Seifert[1]
Bruce F. O'Hara[1†]

[1]Department of Biology, University of Kentucky, Lexington, KY, 40506-0225
[2]Department of Biomedical Engineering, University of Kentucky, Lexington, KY, 40506-0225
[3]Department of Neurology, Washington University School of Medicine, St. Louis, MO, 63110

Running Head: Sleep and activity in spiny mice

[†]To whom correspondence should be addressed: Bruce F. O'Hara, Department of Biology, University of Kentucky, Lexington, KY, 40506-0225. Telephone: (859) 257-2805, Fax: (859) 257-1717, E-mail: bohara@uky.edu

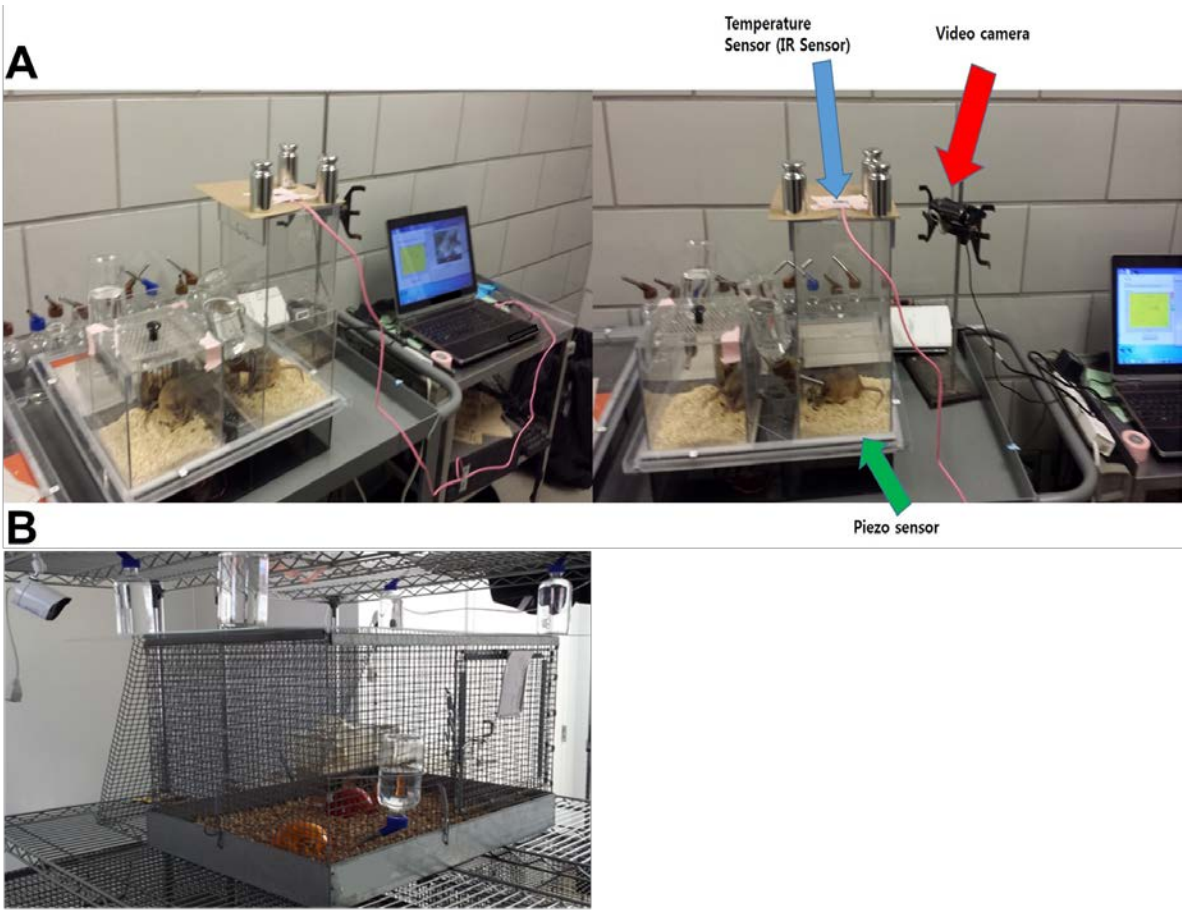

Supplementary figure 1. Experiment setups for piezoelectric system and IR camera recording. (A) Piezoelectric system with IR temperature sensor and video camera, (B) A group cage with four IR camera set-up. The top IR camera covered whole area of bottom to observe the activity of spiny mouse.

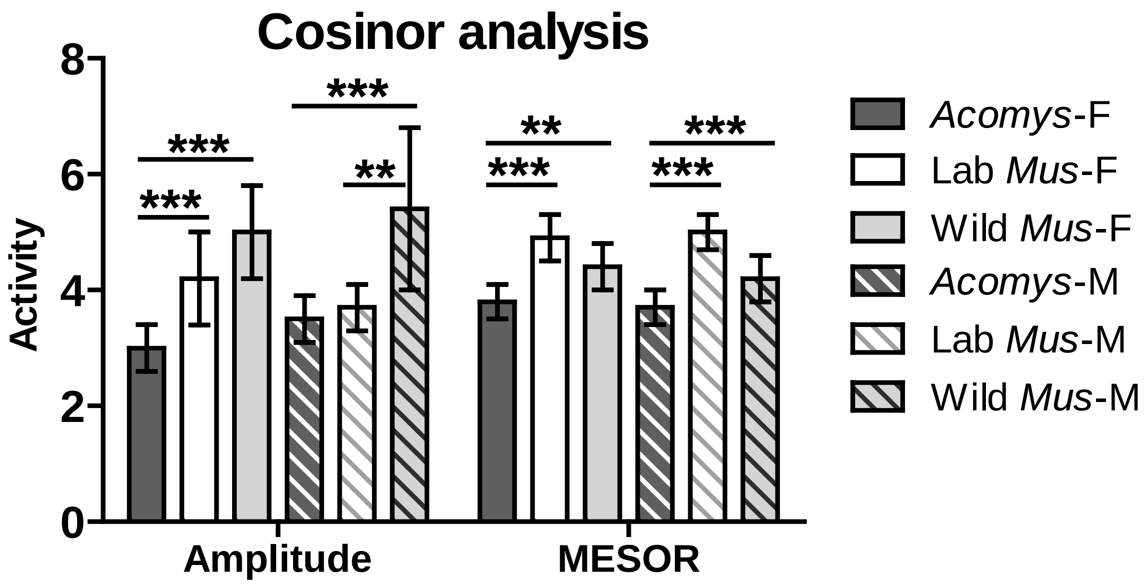

Supplementary figure 2. Cosinor parameters from piezoelectrically determined rhythm of percentage wake. Cosinor analysis shows both species differences and sex differences. One-way ANOVA, Tukey's post-hoc test *P<0.05, **P<0.01, ***P<0.001. (MESOR - midline estimating statistic of rhythm)

		Species		Male		Female	
	Time intervals	*SW Mus*	*BL6 Mus*	*SW Mus*	*BL6 Mus*	*SW Mus*	*BL6 Mus*
Light	6:00-7:00	65.0 ± 8.9	63.4 ± 8.8	71.7 ± 5.4	66.1 ± 7.9	58.4 ± 6.3	60.7 ± 9.3
	7:00-8:00	52.3 ± 6.1	52.8 ± 9.7	52.3 ± 5.8	57.3 ± 8.3	52.2 ± 6.5	48.3 ± 9.2
	8:00-9:00	42.5 ± 5.9	42.7 ± 3.5	42.8 ± 8.3	41.8 ± 3.9	42.2 ± 5.5	43.5 ± 3.0
	9:00-10:00	36.8 ± 3.7	39.7 ± 5.2	36.9 ± 3.8	39.7 ± 4.7	36.7 ± 3.8	39.6 ± 6.0
	10:00-11:00	33.1 ± 3.3	37.4 ± 5.0	32.4 ± 3.6	35.9 ± 4.1	33.8 ± 3.0	38.9 ± 5.5
	11:00-12:00	32.4 ± 3.9	37.3 ± 5.6	31.8 ± 3.8	38.4 ± 4.0	33.0 ± 4.0	36.1 ± 7.0
	12:00-13:00	34.8 ± 5.2	38.7 ± 4.3	35.0 ± 4.3	37.7 ± 3.1	34.5 ± 6.1	39.7 ± 5.2
	13:00-14:00	37.5 ± 6.7	40.2 ± 6.6	38.5 ± 5.7	39.3 ± 7.7	36.4 ± 7.6	41.2 ± 5.7
	14:00-15:00	40.1 ± 9.0	39.4 ± 7.0	42.6 ± 9.0	37.7 ± 6.3	37.7 ± 8.6	41.2 ± 7.6
	15:00-16:00	45.0 ± 8.9	41.7 ± 8.3	48.2 ± 9.5	39.8 ± 9.8	41.8 ± 7.2	43.7 ± 6.4
	16:00-17:00	49.0 ± 7.9	43.5 ± 6.6*	52.6 ± 7.5	45.7 ± 7.3	45.3 ± 6.6	41.3 ± 5.5
	17:00-18:00	63.1 ± 5.4	57.3 ± 4.6*	64.2 ± 5.5	59.7 ± 3.7	62.1 ± 5.3	54.9 ± 4.2
Dark	18:00-19:00	85.8 ± 3.8	82.7 ± 2.5	86.1 ± 3.9	82.5 ± 2.6	85.4 ± 3.9	83.0 ± 2.5
	19:00-20:00	89.7 ± 3.5	90.3 ± 5.8	90.1 ± 3.6	90.5 ± 4.6	89.4 ± 3.6	90.2 ± 7.0
	20:00-21:00	86.0 ± 6.9	87.8 ± 8.7	86.3 ± 6.7	85.8 ± 8.9	85.6 ± 7.4	89.7 ± 6.9
	21:00-22:00	84.3 ± 6.7	87.2 ± 6.9	84.5 ± 5.7	86.8 ± 7.1	84.1 ± 7.7	87.6 ± 7.1
	22:00-23:00	82.7 ± 5.1	83.1 ± 7.5	82.7 ± 5.9	82.7 ± 4.7	82.7 ± 4.5	83.4 ± 9.4
	23:00-0:00	82.2 ± 6.9	82.2 ± 5.4	81.4 ± 8.9	83.4 ± 4.3	83.0 ± 4.3	81.0 ± 6.3
	0:00-1:00	81.1 ± 5.6	85.5 ± 6.0	81.1 ± 7.0	84.8 ± 5.5	81.1 ± 4.1	86.3 ± 6.8
	1:00-2:00	78.6 ± 6.2	83.5 ± 6.7	78.3 ± 7.6	82.3 ± 6.9	78.9 ± 4.8	84.6 ± 6.6
	2:00-3:00	77.3 ± 7.7	75.1 ± 6.0	75.2 ± 9.7	75.3 ± 5.9	79.4 ± 4.4	74.8 ± 6.5
	3:00-4:00	75.4 ± 6.8	72.7 ± 6.0	74.4 ± 7.4	72.8 ± 7.5	76.5 ± 6.3	72.5 ± 4.6
	4:00-5:00	72.1 ± 7.8	73.2 ± 7.4	74.2 ± 7.5	69.5 ± 5.7	70.0 ± 7.8	76.9 ± 7.4
	5:00-6:00	70.8 ± 8.6	74.5 ± 6.3	76.2 ± 5.2	74.8 ± 6.9	64.9 ± 7.3	74.2 ± 6.0*

Supplementary table 1. Piezoelectrically defined sleep amounts (% of total recording time) and sleep bout length (sec) in BL6 Lab *M. musculus* and SW Lab *M. musculus*
